# Cotton flower metabolites inhibit SARS‐CoV‐2 main protease

**DOI:** 10.1002/2211-5463.13477

**Published:** 2022-09-17

**Authors:** Yufang Zhang, Wenkang Li, Yiming Hu, Tianze Ding, Muhammad Mubashar Zafar, Xue Jia, Liya Zhang, Maozhi Ren, Fuguang Li, Wenjing Wang

**Affiliations:** ^1^ Zhengzhou Research Base, State Key Laboratory of Cotton Biology, School of Agricultural Sciences Zhengzhou University China; ^2^ Institute of Cotton Research Chinese Academy of Agricultural Sciences Anyang China; ^3^ Zhengzhou Technology and Business University China; ^4^ Hainan Yazhou Bay Seed Laboratory Sanya China; ^5^ Laboratory of Space Biology, Institute of Urban Agriculture Chinese Academy of Agricultural Sciences Chengdu China; ^6^ Sanya Institute of Zhengzhou University China

**Keywords:** biochemical assay, cotton flower, main protease, SARS‐CoV‐2, variants, virtual screening

## Abstract

Severe acute respiratory syndrome coronavirus 2 (SARS‐CoV‐2) has been spreading globally for over 2 years, causing serious contagious disease and incalculable damage. The introduction of vaccines has slowed the spread of SARS‐CoV‐2 to some extent, but there remains a need for specific and effective treatment. The high chemical diversity and safety profiles of natural products make them a potential source of effective anti‐SARS‐CoV‐2 drugs. Cotton plant is one of the most important economic and medical crops and is the source of a large number of antiviral phytochemicals. In this work, we used SARS‐CoV‐2 main protein (M^pro^) as the target to identify potential anti‐SARS‐CoV‐2 natural products in cotton. An *in vitro* assay showed that of all cotton tissues examined, cotton flower extracts (CFs) exhibited optimal inhibitory effects against M^pro^. We proceeded to use the CF metabolite database to screen natural M^pro^ inhibitors by combining virtual screening and biochemical assays. We identified that several CF natural products, including astragalin, myricitrin, and astilbin, significantly inhibited M^pro^ with half‐maximal inhibitory concentrations (IC50s) of 0.13, 10.73, and 7.92 μm, respectively. These findings may serve as a basis for further studies into the suitability of cotton as a source of potential therapeutics for SARS‐CoV‐2.

## Graphical abstract

Main protein (M^pro^) is highly conserved in SARS‐CoV‐2 and its variants of concern (VOCs, including Alpha, Beta, Gamma, Delta and Omicron) and is a reliable target for screening broad‐spectrum anti‐SARS‐CoV‐2 drugs. Several cotton flower natural products, such as astragalin, myricitrin, and astilbin, exhibited significant inhibition against M^pro^ with half maximal inhibitory concentrations (IC50s) of 0.13, 10.73, and 7.92 μm, respectively.

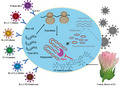

**Abbreviations**
CF: cotton flowers
DMF: *N*,*N*‐dimethylformamide
DMSO: dimethyl sulfoxide
EDTA: ethylenediaminetetraacetic acid
FRET: fluorescence resonance energy transfer
HRV 3C: human rhinovirus 3C protease
IC50: half‐maximal inhibitory concentration
M^pro^
: main protein
NSP: nonstructural protein
PCR: polymerase chain reaction
SARS‐CoV‐2: severe acute respiratory syndrome coronavirus 2
SDS/PAGE: sodium dodecyl sulfate – polyacrylamide gel electrophoresis
VOC: variant of concern

SARS‐CoV‐2, a highly contagious and mutable virus that is responsible for the COVID‐19 (coronavirus disease 2019) pandemic, has caused a global catastrophe since it was first noticed in late 2019 [1]. To date, very few drugs are known to inhibit SARS‐CoV‐2 effectively. The introduction of vaccines has slowed the spread of SARS‐CoV‐2 to some extent. However, billions of persons who are awaiting vaccination and millions of immunocompromised persons who are unlikely to respond robustly to vaccination are still calling for specific and effective treatment [2]. Moreover, many thousands of SARS‐CoV‐2 variants have been identified [3], in which, five variants of concern (VOCs: Alpha, Beta, Gamma, Delta and Omicron) are spreading among global populations. These SARS‐CoV‐2 variants bring enormous challenges for vaccines [4]. Therefore, it remains important to discover broad‐spectrum inhibitors against various SARS‐CoV‐2 variants.

SARS‐CoV‐2 genome encodes a total of 29 proteins: four structural proteins (including envelope, membrane, spike and nucleocapsid), 16 nonstructural proteins (NSPs), and nine accessory proteins [5]. Structural proteins are mainly to form virus particles and infect host cells, while NSPs mostly play important roles in viral replication and other functions. Among the SARS‐CoV‐2 variants, pernicious mutations occur mainly on the spike protein [6]. The NSPs, especially those closely related to novel coronavirus replication, such as PL^pro^ and M^pro^ (proteins essential for SARS‐CoV‐2 replication [7, 8, 9, 10, 11]), are less likely to mutate, which may provide conserved targets for drug discovery [12, 13, 14]. The characterization of SARS‐CoV‐2 M^pro^ crystal structure provides a model for drug discovery [8]. Some studies have obtained several candidate M^pro^ inhibitor through virtual screening [15, 16, 17], which have made great contribution to the screening of anti‐SARS‐CoV‐2 drugs. Nevertheless, it is necessary to verify the real inhibitory effect of the screened M^pro^ inhibitor through biochemical experiments.

Plant‐based natural products have shown excellent antiviral effect against SARS‐CoV‐2 [18, 19]. Cotton (*Gossypium* spp) is a wonderful plant grown for basic needs, such as clothing, food, and shelter. According to Chinese Materia Medica, cotton plant also possesses high medicinal value and health care functions. For example, the extract of cotton flowers (CFs) has been used as an ethical herb by the Uygur people in Xinjiang, China, for a long time to treat mental retardation [20]. Oral CF extract has been reported to be beneficial for age‐related dementia and Alzheimer's disease [21]. In addition, cotton plant can also produce many secondary metabolites with antiviral activity [22, 23]. Gossypin from cotton plant can inhibit herpes simplex viruses [22]. Gossypetin shows a high inhibitory effect on influenza virus *in vitro* [23]. Other metabolites, such as ferulic acid, astragalin, myricitrin, astilbin, kaempferol, and kaempferitrin, also possess certain antiviral properties [24, 25, 26, 27, 28, 29]. Given that cotton plant contains many antiviral products, it is necessary to test whether any of them can inhibit SARS‐CoV‐2.

The current study was designed to identify potential inhibitors against SARS‐CoV‐2 M^pro^ from cotton plant using computational simulation and biochemical assays. The findings of our study may provide some new options for researchers to fight against COVID‐19.

## Materials and methods

### Sequence alignment of M^pro^ in different SARS‐CoV‐2 variants

The M^pro^ protein sequences of original SARS‐CoV‐2 strain (Wuhan‐Hu‐1) as well as the five VOCs, including alpha (B.1.1.7), beta (B.1.351), gamma (P.1), delta (B.1.617.2), and omicron (B.1.1.529), were downloaded from GISAID (https://www.gisaid.org/) and GenBank (http://www.ncbi.nlm.nih.gov/genbank/). The GenBank accession numbers of M^pro^ for Wuhan‐Hu‐1, B.1.1.7, B.1.351, P.1, B.1.617.2 and B.1.1.529 are YP_009725301.1, QUS74317.1, QWW93434.1, QYK37388.1, QRC42887.1 and UKO09917.1, respectively. All five sequences were aligned by dnaman version 8 software (LynnonBiosoft, San Ramon, CA, USA).

### Structural comparison of M^pro^ in the SARS‐CoV‐2 original strain and variants

Sequence alignment revealed that only the M^pro^s in beta (βM^pro^) and omicron variants (οM^pro^) were mutated, we therefore compared the structures of the two mutant M^pro^s with that of the original M^pro^. The M^pro^ structures of original strain and omicron variant were downloaded from Protein Data Bank (PDB) with PDB ID of 6LU7 and 7TVS. The βM^pro^ structure was predicted using alphafold2 [30] via colabfold (version 1.3) [31] with template of original M^pro^ (PBD: 6LU7) [8], MSA mode of “mmseqs2 (Uniref + Environmental)” and pair_mode of “unpaired+paired”. alphafold2 with mmseqs2 was accessible on Google Colab (https://colab.research.google.com/github/sokrypton/ColabFold/blob/v1.3.0/AlphaFold2.ipynb). Other parameters were set as default. The οM^pro^ and predicted βM^pro^ structures were compared with the original M^pro^ and then visualized by the graphics tool pymol version 2.3.2 (Schrödinger, New York, NY, USA).

### Expression and purification of M^pro^

To generate an authentic N‐terminus and C‐terminus of M^pro^ (without additional residues at the termini), our study used a previously reported strategy [8]. The full‐length original SARS‐CoV‐2 M^pro^ gene (ORF1ab polyprotein residues 3264–3569, GenBank code: MN908947.3) was chemically synthesized with codon optimization. Meanwhile, 12 nucleotides coding for the four amino acids AVLQ before the first Ser1 residue were added to form an autocleavage side (AVLQ↓SGFRK) recognized by M^pro^, and 24 nucleotides coding for the eight amino acids GPHis6 at the C‐terminus were added to form a cleavage site (SGVTFQ↓GPHis6) that can be removed by human rhinovirus 3C protease (HRV 3C). Then, the sequence was amplified by polymerase chain reaction (PCR) and inserted into the BamHI and XhoI sites of the pGEX‐6p‐1 plasmid (GE Healthcare, Chicago, IL, USA).

The recombinant plasmid was transformed into *Escherichia coli* BL21 (DE3) cells. First, bacteria were cultured at 2.5 × ***g*** and 37 °C until the OD value reached 1.0. Second, 0.5 mm IPTG was added to the cell culture to induce the expression at 18 °C overnight. Third, the cells were collected by centrifugation at 3000 × ***g*** for 15 min. Fourth, the cell pellets were resuspended in a lysis buffer containing 25 mm Tris (pH 8.0) and 150 mm NaCl, lysed by high‐pressure homogenization, and then centrifuged at 25000 × ***g*** for 40 min. Fifth, the supernatant was hung on a Ni‐NTA affinity column (Qiagen, Shanghai, China) and washed twice with resuspension buffer containing 20 mm imidazole. Sixth, the collected protein was eluted by cleavage buffer (50 mm Tris–HCl pH 7.0, 150 mm NaCl) including 300 mm imidazole. Finally, the target protein was digested with Human rhinovirus 3C protease overnight to remove the C‐terminal His‐tag. The concentrated target protein was stored in using 10 mm Tris–Hcl (pH 7.5) buffer.

### Enzymatic activity assays

The activity of recombinant M^pro^ was evaluated by using the fluorescence resonance energy transfer (FRET) substrate MCA‐AVLQSGFR‐Lys(Dnp)‐Lys‐NH2 [32]. The amino acid sequence of the FRET substrate was derived from the N‐terminal self‐cleavage sequence of M^pro^. The fluorescence intensity was recorded by a Fluoraskan Ascent Fluorometer (Thermo, Waltham, MA, USA) with excitation and emission wavelengths of 320 and 405 nm, respectively. The reaction buffer contained 50 mm Tris–HCl (pH 7.3), 1 mm ethylenediaminetetraacetic acid (EDTA), and 30 nm M^pro^. For enzyme kinetics studies, the fluorescence intensity was recorded as soon as the FRET substrates (different concentrations ranging from 0 to 50 μm) were added to the reaction buffer. The initial velocity of each reaction was calculated by linear regression for the first 10 min of the kinetic progress curves. Finally, the kinetic parameters *km* and *Vmax* were obtained by plotting the initial velocity against the substrate concentration using the Michaelis–Menten equation in graphpad prism 8 software (GraphPad Software, San Diego, CA, USA).

### Preparation of extracts from various tissues of cotton

Zhongmian‐24 (ZM24) from the Institute of Cotton Research, the Chinese Academy of Agricultural Sciences (CAAS), was used in this work. The cotton seeds were planted in the experimental field at the School of Agricultural Sciences, Zhengzhou University, China. After 90 days, different tissues, including flowers, leaves, roots, stems, and cotton bolls, were collected from the cotton plants at the same growth states. A total of 0.3 g fresh weight of each tissue was ground thoroughly to powder in liquid nitrogen and then ultrasound‐treated for 3 h in 1 mL *N*,*N*‐dimethylformamide (DMF; Aladdin) [33]. The extracts were centrifuged at 10 000 × ***g*** for 5 min. The supernatants were collected and stored at −20 °C until further use.

### Enzyme inhibition assay of cotton extracts on SARS‐CoV‐2 M^pro^

For the enzyme inhibition assay, each cotton extract (final concentration of 15 mg·mL^−1^) was incubated with 0.2 μm SARS‐CoV‐2 M^pro^ at 37 °C for 30 min in reaction buffer containing 50 mm Tris–HCl (pH 7.3) and 1 mm EDTA. When the FRET substrate (final concentration of 20 μm) was added to the reaction, the fluorescence intensity was immediately monitored every 30 s for 1 h. DMSO was used as the negative control for the whole study. The inhibition rate was calculated by the ratio of the initial velocity.

### Enzyme inhibition assay of CF metabolites on SARS‐CoV‐2 M^pro^

The enzyme inhibition assay of CF metabolites against SARS‐CoV‐2 M^pro^ was performed by treating recombinant M^pro^ with different CF metabolites (astragalin, myricitrin, astilbin, kaempferitrin, and kaempferol) followed by the addition of fluorescence resonance energy transfer (FRET) substrate. For astragalin, myricitrin, and astilbin, the final concentrations were varied from 0 to 20, 0 to 5000, and 0 to 10 000 μm, respectively. The experimental conditions were the same as that for cotton extracts on SARS‐CoV‐2 M^pro^. The IC50 was calculated by plotting the initial velocity against various concentrations of inhibitors by graphpad prism 8 software. In addition, 10 μm kaempferitrin and kaempferol were used to compare their inhibitory effects against M^pro^.

### Virtual screening of SARS‐CoV‐2 M^pro^ inhibitors

The in‐house CF metabolome in our laboratory was used to identify natural SARS‐CoV‐2 M^pro^ inhibitors. First, the CF metabolite database was established by obtaining the 3D structures downloading from the Chemicalbook website or drawing with chem3d software (PerkinElmer, Waltham, MA, USA). Then, virtual screening was performed with Autodock vina in pyrx 0.8 [34] by using pre‐processed SARS‐CoV‐2 M^pro^ (6LU7) as the receptor and the small molecules in the CF metabolite database as the ligands. The grid box centered for M^pro^ was *X* = −11.82, *Y* = 14.94, and *Z* = 67.92 and the dimensions of the grid box were set as 65 × 65 × 65 Å. The spacing value was the default value of 0.375 Å. Other docking parameters were kept at their default. The docking result was analyzed and visualized in pymol and chimerax 1.2.5 software (University of California, Los Angeles, CA, USA). The 2D interactions of small molecules and M^pro^ were analyzed by the online tool proteins plus (https://proteins.plus).

## Results

### Sequence alignment and structural comparison of M^pro^ in different SARS‐CoV‐2 variants

**Fig. 1:**
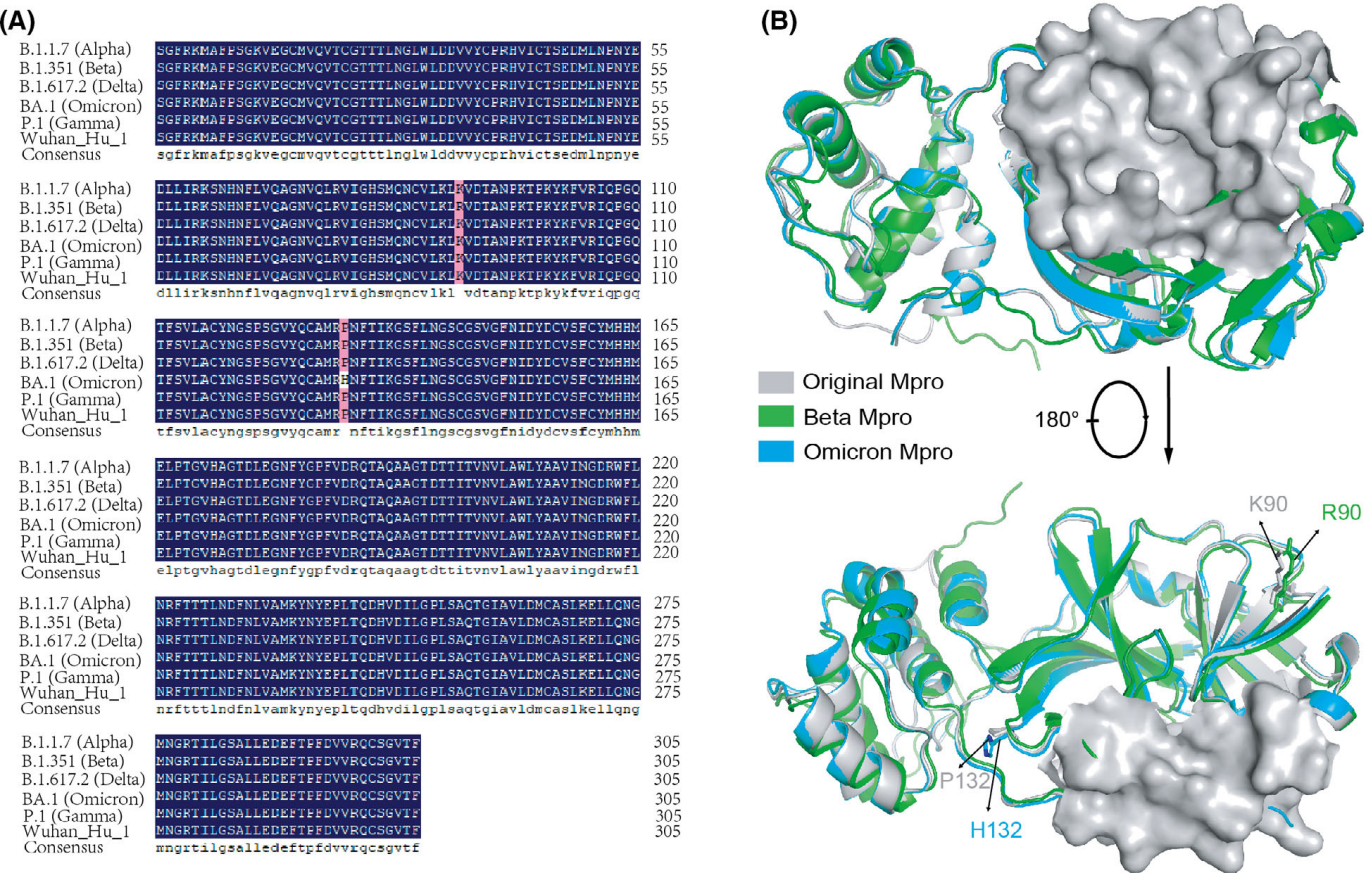
Conservation analysis of M^pro^ in five dominant SARS‐CoV‐2 variants. (A) Sequence alignment of SARS‐CoV‐2 M^pro^ in the original strain of Wuhan‐Hu‐1 and five dominant variants of alpha, beta, gamma, delta, and omicron. The amino acid color in dark blue indicates 100% conservation. (B) Structural comparison of the original M^pro^ with variant M^pro^s in the beta variant (βM^pro^) and omicron variant (οM^pro^). The surface shown in gray represents the active pocket of M^pro^. The amino acids of K90 and P132 in the original M^pro^, R90 in βM^pro^, and H132 in οM^pro^ are shown with sticks.

SARS‐CoV‐2 has mutated thousands of times and several dominant variants have emerged, including alpha (B.1.1.7), beta (B.1.351), gamma (P.1), delta (B.1.617.2), and omicron (B.1.1.529), so it is necessary to identify a drug target that is conserved in different variants. The sequences of the five dominant variants were aligned by DNAman. There was almost no mutation in these variants except for one mutation of K90R in beta variants and one mutation of P132H in omicron variants (Fig. 1A). In addition, these mutations are far from the active center of M^pro^ (Fig. 1B), which has no effect on the structure of the active center. Therefore, M^pro^ of SARS‐CoV‐2 can be used as a reliable target for drug discovery against all the dominant variants.

### Enzymatic activity for recombinant SARS‐CoV‐2 M^pro^

**Fig. 2:**
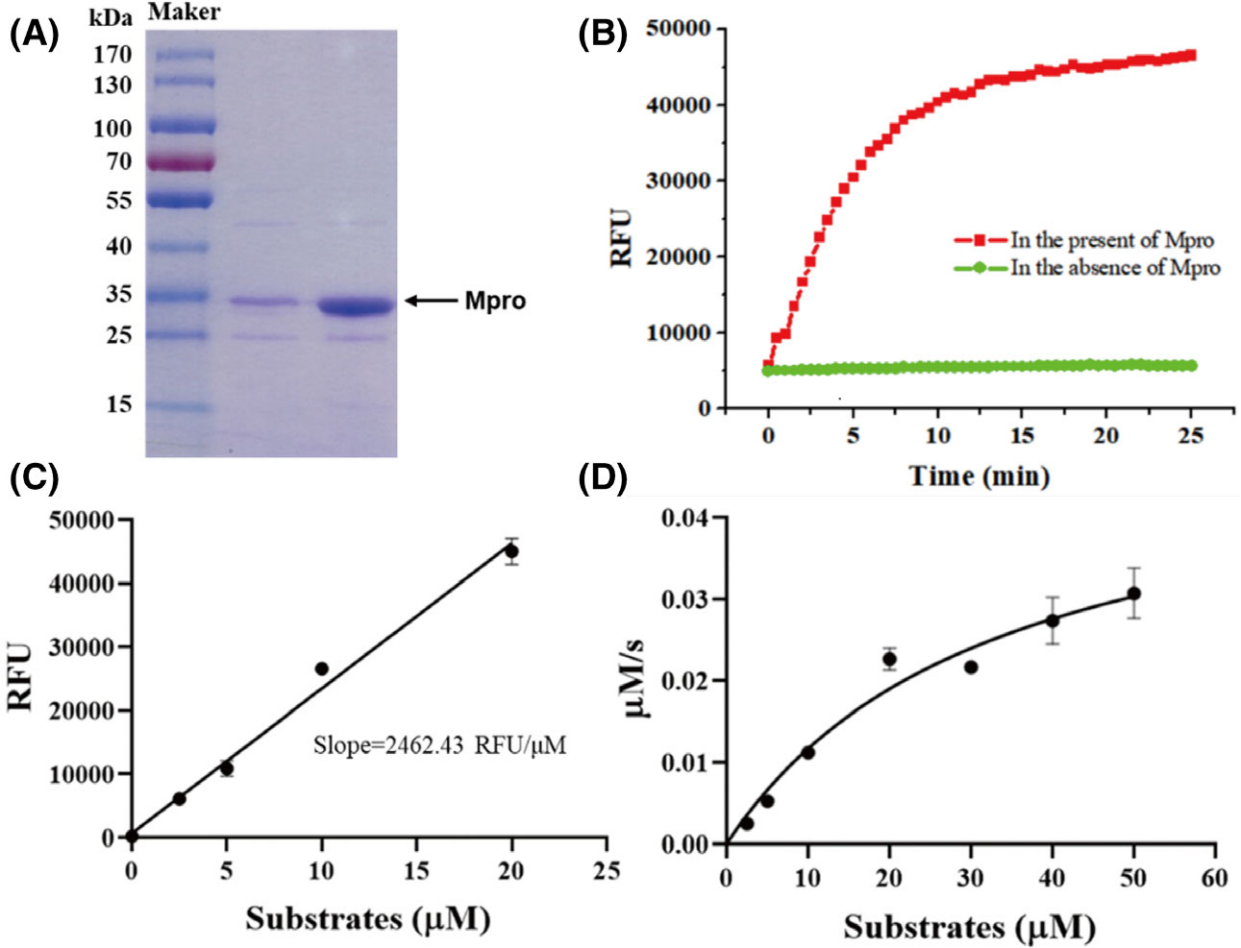
SARS‐CoV‐2 M^pro^ expression and characterization. (A) SDS/PAGE of recombinant M^pro^ (lane 1–2). (B) The relative fluorescence unit (RFU) of FRET substrates with (red) or without (green) recombinant M^pro^. (C) A standard curve converts RFU to the amount of the cleaved substrates. Error bars are the mean ± SD (*n* = 3). (D) Michaelis–Menten plot of recombinant M^pro^ with various concentrations of FRET substrates. Error bars are the mean ± SD (*n* = 3).

Recombinant SARS‐CoV‐2 M^pro^ with native N and C termini was obtained according to a method reported previously [8]. The molecular weight of M^pro^ was approximately 33 kDa according to sodium dodecyl sulfate – polyacrylamide gel electrophoresis (SDS/PAGE; Fig. 2A). To characterize its enzymatic activity, a FRET assay was performed with a fluorescently labeled substrate, MCA‐AVLQ↓SGFR‐Lys(Dnp)‐Lys‐NH2 [32]. In the presence of M^pro^, the fluorescence intensity increased rapidly at first and then gradually reached a plateau, whereas little change was observed in the absence of protein (Fig. 2B). To study the enzyme kinetics, a standard curve was generated to convert the relative fluorescence unit (RFU) to the amount of the cleaved substrate (Fig. 2C). Next, the enzymatic activity of this SARS‐CoV‐2 M^pro^ was characterized by measuring the parameters *km*, *Kcat*, and *Vmax*. When 200 nm M^pro^ was mixed with different concentrations of FRET substrates (0–50 μm), the initial velocity was measured and plotted against substrate concentration using the Michaelis–Menten equation in graphpad prism 8 (Fig. 2D). The best‐fit values of *km* and *Vmax* were 33.12 μm and 0.05 μm·s^−1^, respectively. The calculated *kcat*·*km* ^−1^ was 7608.69 s^−1^·m ^−1^, suggesting that the obtained M^pro^ are active enough for further research.

### The cotton flower extract strongly inhibits SARS‐CoV‐2 M^pro^

To test whether the cotton plant extracts possess inhibition potential against SARS‐CoV‐2, we prepared extracts of different cotton tissues, such as flowers, bolls, leaves, roots, and stems. The inhibitory ratios of the extracts at the same concentrations against SARS‐CoV‐2 M^pro^ are shown in Fig. S1. The crude extract of the cotton flower exhibits a significant inhibitory effect against M^pro^, suggesting that cotton flower contains some candidate inhibitory ingredients that can inhibit SARS‐CoV‐2 M^pro^.

### Virtual screening of SARS‐CoV‐2 M^pro^ inhibitors based on the metabolite database of cotton flower

To identify the natural products that inhibit SARS‐CoV‐2 M^pro^ in cotton flowers, we established a metabolite database according to the in‐house CF metabolome data in our laboratory (data not yet published). A total of 228 metabolites were identified (Fig. S2A, Table S1), including 89 flavonols, 60 flavonoids, 33 isoflavones, 11 anthocyanins, etc, of which 131 metabolites have well‐defined three‐dimensional structures and can be used for virtual screening. Virtual screening was carried out with CF metabolites as ligands and SARS‐CoV‐2 M^pro^ (PDB: 6LU7) as the receptor. As shown in Fig. S2B, all the ligands can bind to the active pocket of M^pro^ with docking scores less than −5.5 kcal·mol^−1^. Moreover, most of the metabolites exhibited docking scores less than −7.0 kcal·mol^−1^, indicating their great inhibitory potentials against SARS‐CoV‐2 M^pro^.

### Predicted binding poses of CF metabolites to SARS‐CoV‐2 M^pro^

**Fig. 3:**
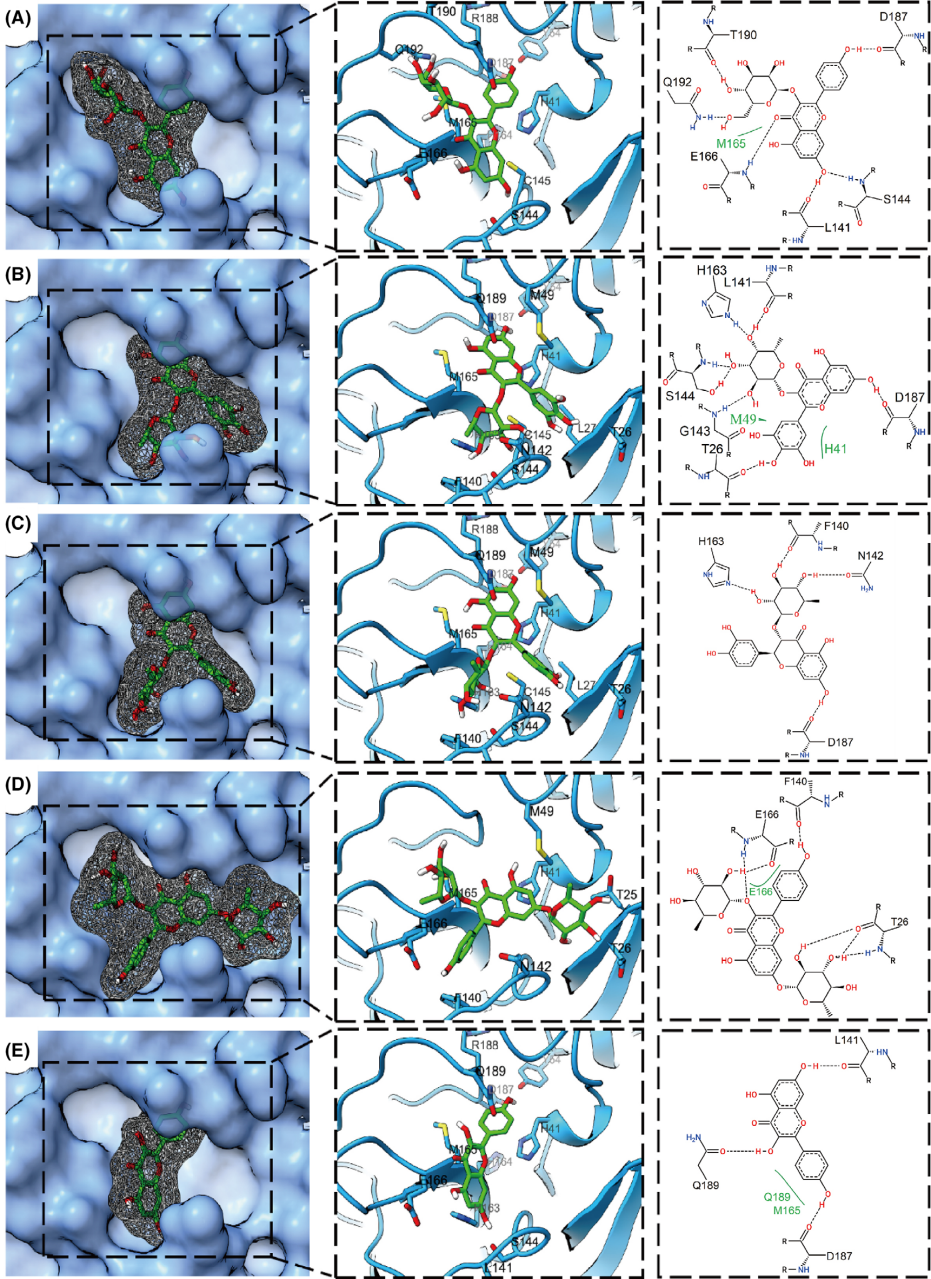
Binding poses of six CF metabolites with SARS‐CoV‐2 M^pro^. (A–E) Three views of astragalin (A), myricitrin (B), astilbin (C), kaempferitrin (D), and kaempferol (E) binding with SARS‐CoV‐2 M^pro^ (PDB: 6LU7). SARS‐CoV‐2 M^pro^ is represented by the blue surface (left), blue illustration (middle), and 2D amino acid residues (right). CF metabolites are represented by green sticks (left), green sticks with gray mesh (middle), and 2D structures (right). The dashed black lines and solid green lines in the right figures represent hydrogen bonds and hydrophobic interactions, respectively.

As reported, the CF metabolites astragalin, myricitrin, astilbin, kaempferitrin, and kaempferol have broad‐spectrum antiviral activity [24, 25, 26, 27, 28, 29], so these five metabolites were researched further. Astragalin could most strongly bind to M^pro^ with the lowest docking score of −9.4 kcal·mol^−1^ through many favorable interactions, such as hydrogen bonds (H‐bonds) and hydrophobic interactions (Fig. 3A). Specifically, astragalin forms H‐bonds with the main chains of L141, S144, C145, E166, D187, T190, etc, and the side chains of S144, Q192. In addition, hydrophobic interactions also exist between astragalin and residues H41, M165, and Q189 of M^pro^. The docking scores of myricitrin and astilbin against M^pro^ are −9.2 and −9.1 kcal·mol^−1^, respectively. Myricitrin binds to M^pro^ by 7 H‐bonds with L141, H163, S144, G143, T26, and D187, as well as two hydrophobic interactions with M49 and H41 (Fig. 3B). For astilbin, the interactions against M^pro^ are mainly H‐bonds formed with H163, F140, N142, and D187 (Fig. 3C). Kaempferitrin, another classic antiviral molecule, can also bind tightly to M^pro^ with a binding score of −8.5 kcal·mol^−1^ by several H‐bonds and hydrophobic interactions against F140, T26, and E166 (Fig. 3D). As a comparison, kaempferol, the aglycon of kaempferitrin, was also used to evaluate the binding ability against M^pro^. Slightly weaker interactions between kaempferol and M^pro^ are observed with a binding score of −7.8 kcal·mol^−1^ by 3 H‐bonds and 2 hydrophobic interactions (Fig. 3E). In total, the binding of the above molecules can surpassingly block the active pocket and cover the catalytic residues H41 and C145 of M^pro^, indicating their excellent inhibition potentials against M^pro^.

### Strong inhibition of CF metabolites against SARS‐CoV‐2 by *in vitro* assay

**Fig. 4:**
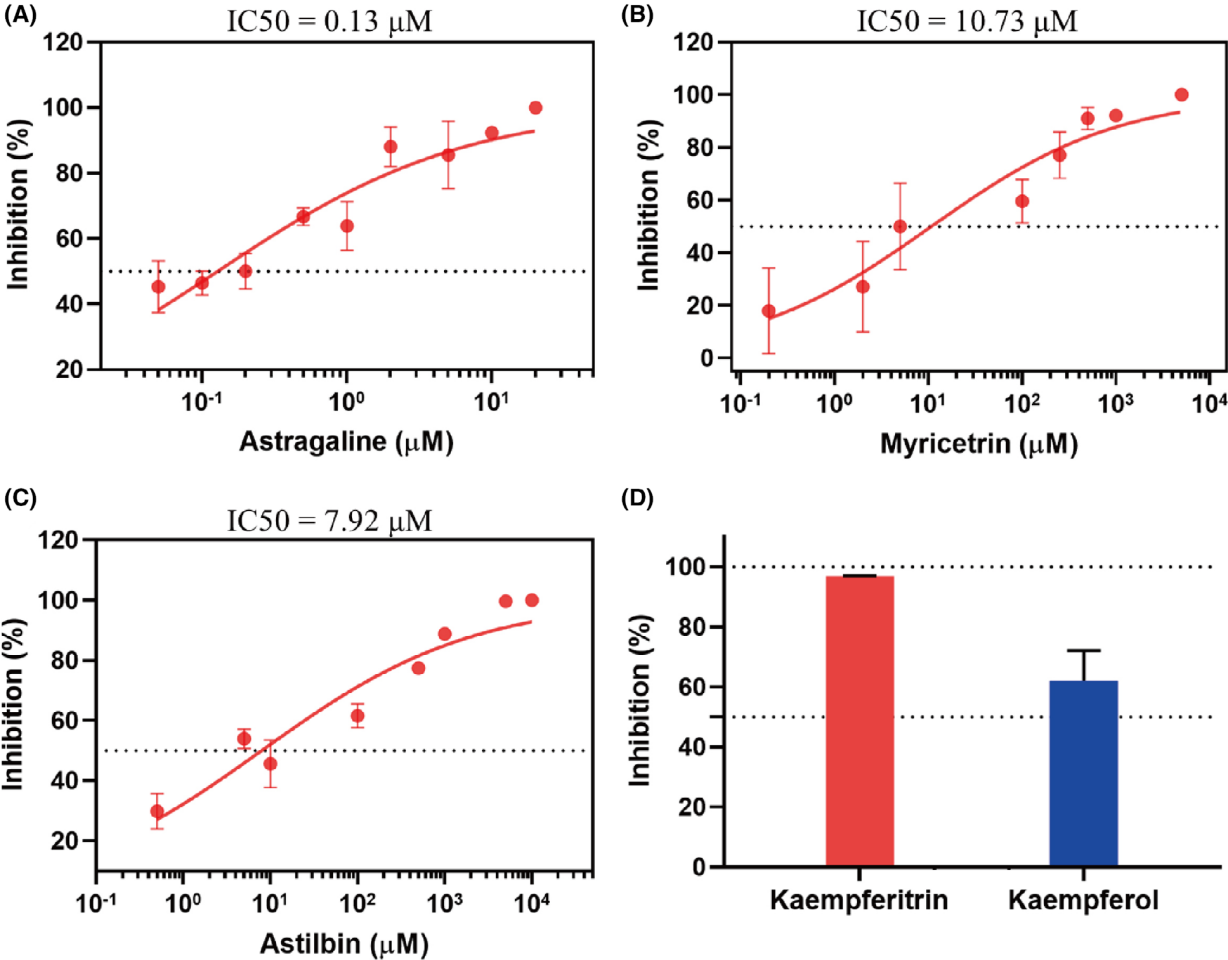
Inhibition of SARS‐CoV‐2 M^pro^ by six CF metabolites. (A–C) the *in vitro* inhibition curves of SARS‐CoV‐2 M^pro^ by astragalin (A), myricitrin (B), and astilbin (C). (D) The inhibition rates of SARS‐CoV‐2 M^pro^ by 10 μm kaempferitrin (red) and kaempferol (blue). The data are the means ± standard deviation (SD) of three repeats.

We tested the inhibitory activity of the above five major ingredients from cotton flower: astragalin, myricitrin, astilbin, kaempferitrin, and kaempferol. Among the metabolites, the strongest inhibition of SARS‐CoV‐2 M^pro^ was observed for astragalin, with a lowest half‐maximal inhibitory concentration (IC50) of 0.13 μm (Fig. 4A). The IC50 values were 10.73 μm for myricitrin and 7.92 μm for astilbin (Fig. 4B,C), which were slightly higher than the IC50 value of astragalin. The inhibitory activity of kaempferol against M^pro^ was slightly less than the inhibitory activity of kaempferitrin (Fig. 4D). At a final concentration of 10 μm, kaempferol inhibited M^pro^ by 96.81%, whereas the inhibition ratio of kaempferol was only 62.05%. In total, the above compounds showed high inhibitory activities against SARS‐CoV‐2 M^pro^, especially astragalin, myricitrin and astilbin, indicating their excellent inhibitory potentials on the replication of SARS‐CoV‐2.

## Discussion

SARS‐CoV‐2 is still spreading and mutating and is likely to coexist with humans for a long time. Improving the daily diet and consuming more functional foods containing natural SARS‐CoV‐2 inhibitors may be an effective means for preventing SARS‐CoV‐2. In this work, SARS‐CoV‐2 M^pro^ is selected as the target for natural inhibitor screening for its high conservation in different mutant strains. Through theoretical screening and enzymological verification, we found that astragalin, myricitrin, astilbin, kaempferitrin, and kaempferol in cotton flowers showed significant inhibition against SARS‐CoV‐2 M^pro^, indicating that cotton flower may be an effective natural material for inhibiting SARS‐CoV‐2.

Generally, studies on cotton plant have mainly focused on its agronomic properties, such as fiber and oil, and have often ignored its medicinal and edible value. Cotton flowers are rich in flavonoids, such as quercetin, isoquercetrin, and quercimeritrin. Interestingly, most of these flavonoids have a broad spectrum of antiviral activity. Astragalin, for instance, shows great antihepatitis C virus [25] and anti‐influenza activity [35]. Myricitrin, in addition to inhibiting influenza virus, can also inhibit coxsackie A16 viruses [26]. Astilbin also possesses antiviral properties against human immunodeficiency virus‐1 [27] as well as bovine and equine herpesviruses [36]. In addition, kaempferitrin and its aglycone kaempferol can inhibit a series of viruses, such as Dengue virus‐2 [37], pseudorabies virus [28], and bovine herpesvirus 1 [29]. Since the SARS‐CoV‐2 outbreak, although many studies have indicated the inhibitory potential of some substances against M^pro^ by virtual screening, few of them have been validated by enzymic methods. In this work, based on the great inhibitory activity of cotton flowers against SARS‐CoV‐2 M^pro^, we selected and verified several natural inhibitors of M^pro^, that is, astragalin, myricitrin, astilbin, kaempferitrin, and kaempferol. However, it remains to further study whether these M^pro^ inhibitors can surpassingly prevent the replication of SARS‐CoV‐2.

Continuous variation in SARS‐CoV‐2 brings great difficulties to epidemic prevention. Considering that cotton flowers are rich in natural products that effectively inhibit highly conserved M^pro^, which might be processed as a functional food for daily use to prevent various SARS‐CoV‐2 variants.

## Conflict of interest

The authors declare no conflict of interest.

## Author contributions

MR, FL and WW conceived and designed the project; WL, YZ, TD and WW acquired the data; XJ provided the resources; WL, TD, YH and WW analyzed and interpreted the data; LZ, MZ, MR, FL and WW wrote and reviewed the paper; WL, TD and WW revised the paper. All authors have read and agreed to the published version of the manuscript.

## Supporting information


**Fig. S1.** The *in vitro* anti‐SARS‐CoV‐2 M^pro^ activity of cotton extracts. Error bars are the mean ± SD (*n* = 3).
**Fig. S2.** Virtual screening of M^pro^ inhibitors based on CF metabolites. (A) The statistics and classification for all identified metabolites (blue) or that with well‐defined three‐dimensional structures (red) in CF tissue. (B) The statistics for binding energies of the CF metabolites to the SARS‐CoV‐2 M^pro^. The list of CF metabolites and corresponding binding energies are shown in Table S1.

**Table S1.** The list of CF Compounds and their binding scores with SARS‐CoV‐2 M^pro^.

## Data Availability

All data generated or analyzed during this study are included in this published article (and its supporting information files). Samples of the compounds can be available from the authors.
