# Supplementary material for: Cotton flower metabolites inhibit SARS‐CoV‐2 main protease
Source: FEBS Open Bio. 2022 Sep 17;12(10):1886–95. doi: 10.1002/2211-5463.13477 (PMC9527594; doi:10.1002/2211-5463.13477)
Supplement: Supplementary file 1 — Fig. S1. The in vitro anti‐SARS‐CoV‐2 Mpro activity of cotton extracts. Error bars are the mean ± SD (n = 3). Fig. S2. Virtual screening of Mpro inhibitors based on CF metabolites. (A) The statistics and classification for all identified metabolites (blue) or that with well‐defined three‐dimensional structures (red) in CF tissue. (B) The statistics for binding energies of the CF metabolites to the SARS‐CoV‐2 Mpro. The list of CF metabolites and corresponding binding energies are shown in Table S1. [file FEB4-12-1886-s001.docx]

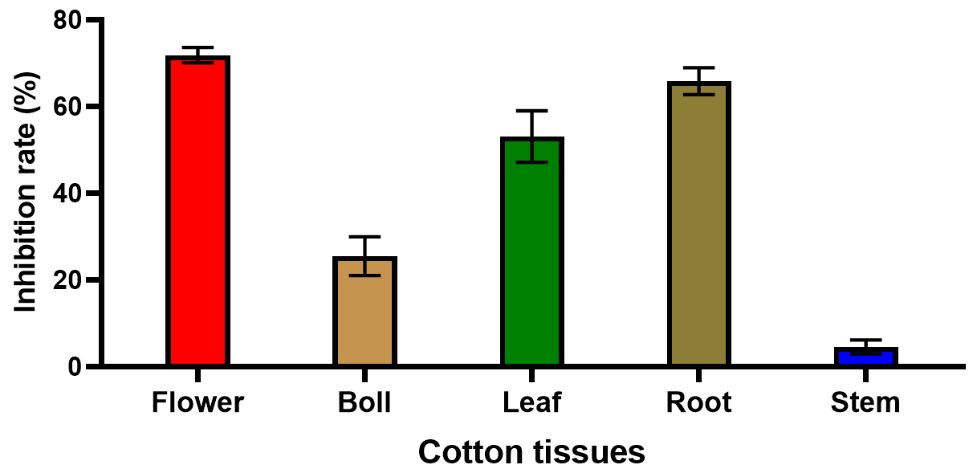


**Fig S1.** The in vitro anti-SARS-CoV-2 M^pro^ activity of cotton extracts. Error bars are the mean ± s.d. (n=3).


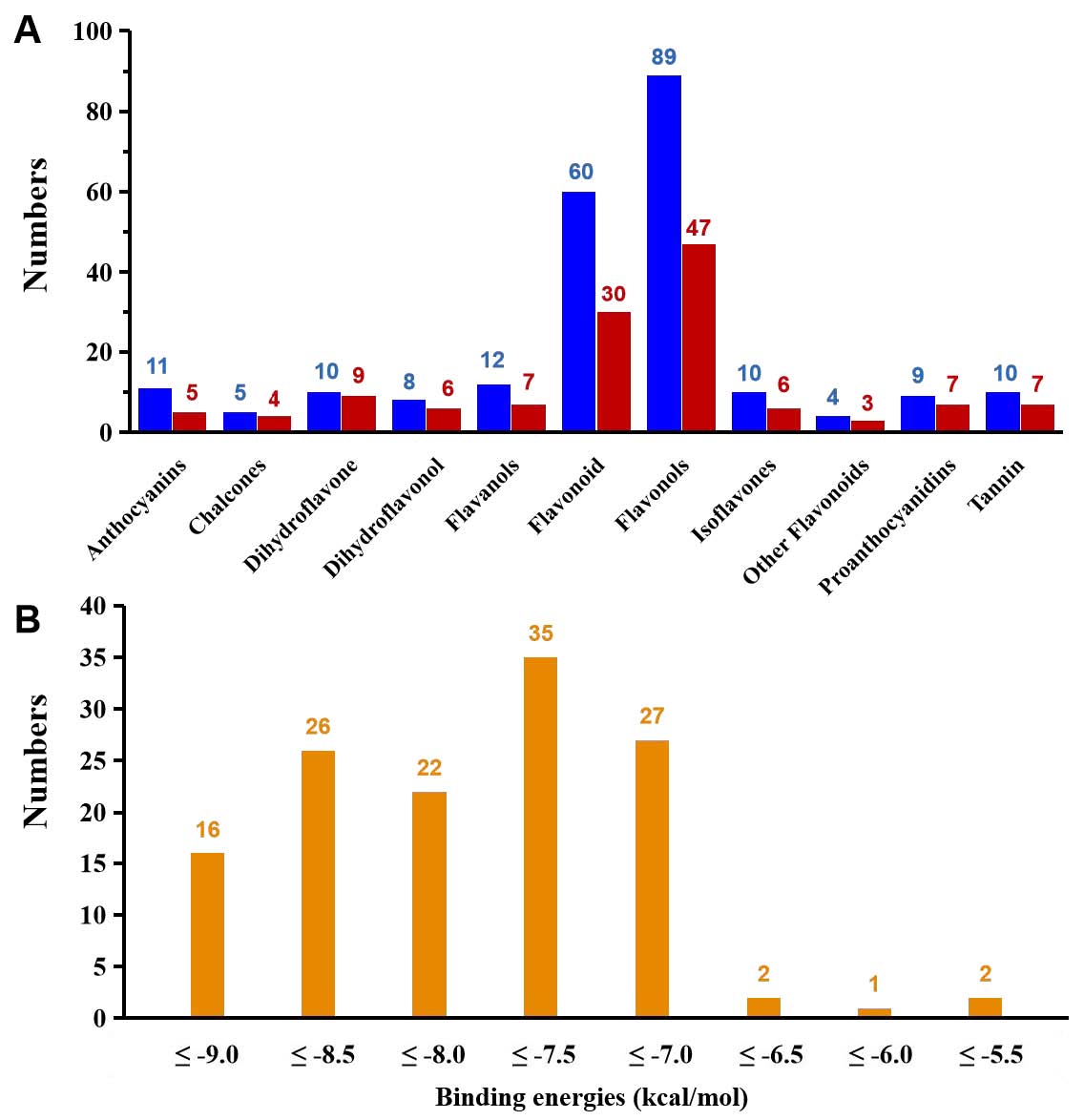


**Fig S2.** Virtual screening of M^pro^ inhibitors based on CF metabolites. (**A**) The statistics and classification for all identified metabolites (blue) or that with well-defined three-dimensional structures (red) in CF tissue. (**B**) The statistics for binding energies of the CF metabolites to the SARS-CoV-2 M^pro^. The list of CF metabolites and corresponding binding energies are shown in Supplementary Table S1**.**
